# The effect of perceived teachers’ interpersonal behavior on students’ learning in physical education: a systematic review

**DOI:** 10.3389/fpsyg.2023.1233556

**Published:** 2023-08-29

**Authors:** Lijun Tian, Jun Shen

**Affiliations:** ^1^College of Education, Zhejiang Normal University, Jinhua, China; ^2^College of Education, Nanchang Normal University, Nanchang, China; ^3^College of Physical Education and Health Sciences, Zhejiang Normal University, Jinhua, China

**Keywords:** teachers’ interpersonal behavior, students’ learning, learning motivation, physical activity level, learning emotion, physical education

## Abstract

The primary purpose of this study was to conduct the first systematic review on teachers’ interpersonal behaviors and students’ learning within the physical education context. We searched the English literature in the EBSCOhost, Web of Science, SCOPUS and PubMed electronic databases and following screening, data extraction, quality assessment, 35 published articles were included in this review. The results showed that: perceived teachers’ supportive behaviors have effect on autonomous motivation of students, and perceived teachers’ controlling behaviors have effect on students’ controlling motivation and amotivation, the influencing mechanism may be that teachers’ interpersonal teaching behaviors make students’ basic psychological needs satisfaction or frustration; perceived teachers’ international behaviors have effect on PE learning emotion of students, and perceiving teachers’ supportive behaviors can trigger students’ positive learning emotion, on the contrary, perceiving teachers’ controlling behaviors can stimulate students’ negative learning emotion; teachers’ interpersonal behaviors have effect on students’ psychological well-being and physical activity levels in class with the evidence insufficient, and more evidence are needed. It is extremely necessary to expand such study direction and strengthen better quality study to explore the effect of teachers’ interpersonal behaviors on learning process and learning outcome in physical education class, and examine the indirect influence mechanism through that the relationship is connected. The present review provides preliminary evidence to enhance the quality of physical education teaching in class, promote students’ academic performance by intervening teachers’ interpersonal behaviors.

## 1. Introduction

The health benefits of a physically active lifestyle during adolescence have been well demonstrated. However, according to the report “Global trends in insufficient physical activity among adolescents: a pooled analysis of 298 population-based surveys with 1.6 million participants,” published in Lancet Child Adolescent Health in 2019, worldwide, 81.0% of students aged 11–17 years were not sufficiently active, including 77.6% of boys and 84.7% of girls (Guthold et al., 2019). The majority of adolescent students do not fulfill the recommendations of the World Health Organization, that teenagers engage in at least 60 min of moderate-to-vigorous intensity physical activity (MVPA) every day (WHO, 2020), which is compromising their health. Physical inactivity has become one of the four principal causes of human death and is closely associated with cardiovascular risk factors and obesity problems among children and adolescents. Therefore, enhancing physical activity has become an urgent public health concern for children and adolescents. For this age group, physical education (PE) is the key to developing physical fitness, which not only requires that students be active during the school day but also allows them to gain information, skills, and experience and to develop positive physical exercise habits engaged outside of school. Nonetheless, many adolescent students lack a positive attitude, have poor motivation, and display no interest in physical activity in PE classes. Researches reveal that the time students spend in MVPA does not meet the US Center for Disease Control and Prevention or United Kingdom Associations for PE recommendation of 50% in PE classes in elementary and secondary schools (Hollis et al., 2015, 2017). Some studies have shown that promotion of pupils’ physical exercise and motivation is substantially aided by PE teachers (Moreno-Murcia et al., 2018; Emeljanovas et al., 2020; Van Doren et al., 2021).

Researches on teachers’ behavior focus on two aspects. On the one hand, specific teaching behaviors are studied from a pedagogical viewpoint, such as teachers**’** questioning, teaching, instructing, and managing strategies. On the other hand, from the perspective of interpersonal interactions, teaching behaviors are regarded as interpersonal interaction behaviors between teachers and students, such as encouragement, comfort, and criticism. Teachers**’** behavioral characteristics determine the nature of teacher-student relationships to some extent, and good interpersonal skills and behaviors can create a positive environment for students**’** development. According to Wubbels et al. (1991), it is most crucial to study teacher behavior from an interpersonal perspective, and they conducted extensive research on the topic.

A model exists for interpersonal teacher behavior in which interpersonal teaching behavior is mapped using two independent dimensions: the influence dimension and the proximity dimension (Wubbels et al., 2006). One dimension involves the extremes of dominance and submission; the other dimension involves the extremes of cooperation and opposition. Based on these two dimensions, teacher behaviors are classified into eight categories: leadership, helpful/friendly, understanding, giving students freedom, uncertain, dissatisfied, admonishing, and strict (Korthagen and Evelein, 2016). This review focuses specifically on teachers’ interpersonal behavior in PE.

To date, empirical research has shown how teachers’ interpersonal behaviors affect students’ learning in PE. In general, researchers have explored the effects on students’ PE learning primarily from two aspects: teachers’ supportive behaviors and teachers’ controlling behaviors (Moreno-Murcia et al., 2018; Coterón et al., 2020; Burgueño et al., 2021), with the majority referring to positive behaviors. Numerous studies have revealed that perceived supportive actions by teachers have a favorable influence on students’ motivation, positive emotion, behavioral engagement, physical activity, and other PE learning outcomes (Hagger et al., 2009; Hein and Caune, 2014; Moreno-Murcia et al., 2018; Coterón et al., 2020; Emeljanovas et al., 2020; Van Doren et al., 2021; Petiot and Desbiens, 2022). Although teachers’ controlling behavior in PE is less explored, it usually relates to students’ negative learning outcomes; for example, students’ motivation has been inversely correlated with teachers’ controlling behaviors, which explained physical activity intention (De Meyer et al., 2014; Koka and Sildala, 2018; Moreno-Murcia et al., 2018) as well as feelings of anger and bullying behavior (Hein et al., 2015). Therefore, teachers’ controlling behaviors cannot be ignored. We need to investigate not only the impact of positive teacher behaviors on student performance in PE learning, but also the impact of negative teacher behaviors on students’ academic outcomes, which are equally important.

A few reviews exist on teacher behavior from an interpersonal interaction perspective, while only one review has been found in PE settings (Hein, 2012); however, it is a theoretical review published a decade ago. This study provides the first systematic review of research on teachers’ interpersonal behaviors and students’ learning outcomes in PE. What teachers do in class has an important impact on students’ learning. The review will identify potential instructional improvements in PE classes. To understand the effect on students’ learning results, we could intervene PE teachers to make them improve the teaching practice, so that promote the level of physical activity of students.

## 2. Method

Reporting in the present review aligns with the Preferred Reporting Items for Systematic Reviews and Meta-Analyses (PRISMA) statement (Page et al., 2021).

### 2.1. Information source

We conducted systematic searches in the electronic databases EBSCOhost, Web of Science, SCOPUS, and PubMed using various combinations of two groups of subject words.

### 2.2. Search strategy

In December 2022, the literature was searched using different combinations of the following two groups of subject terms: (1) “teacher* behavior*,” OR “teaching behavior*” OR “teacher* interpersonal behavior*;” (2) “physical education” OR “physical learning” OR “sport learning” OR “physical education learning.” The retrieved literature was sequentially screened by title and abstract, and the whole text was extracted for evaluation. Finally, a manual search was carried out for the included references to supplement studies missed by the electronic databases. The retrieval was performed independently by two researchers.

### 2.3. Eligibility criteria

This systematic review adopted the following inclusion criteria: (a) included a sample of children or adolescents excluding infants, adults, animals, special populations, and persons with disabilities; (b) written in English and published in peer-reviewed journals, excluding non-English literature, unpublished literature, conference abstracts, conference literature, dissertations, and books; (c) included empirical studies, excluding reviews, and comments; (d) included statistical evaluation of the link between at least two of these constructs: teachers’ supporting behaviors, teachers’ controlling behaviors, or students’ learning outcomes, including cognitive, affective, or behavioral outcomes; and (e) conducted in the PE class setting.

### 2.4. Selection process

**Figure 1:**
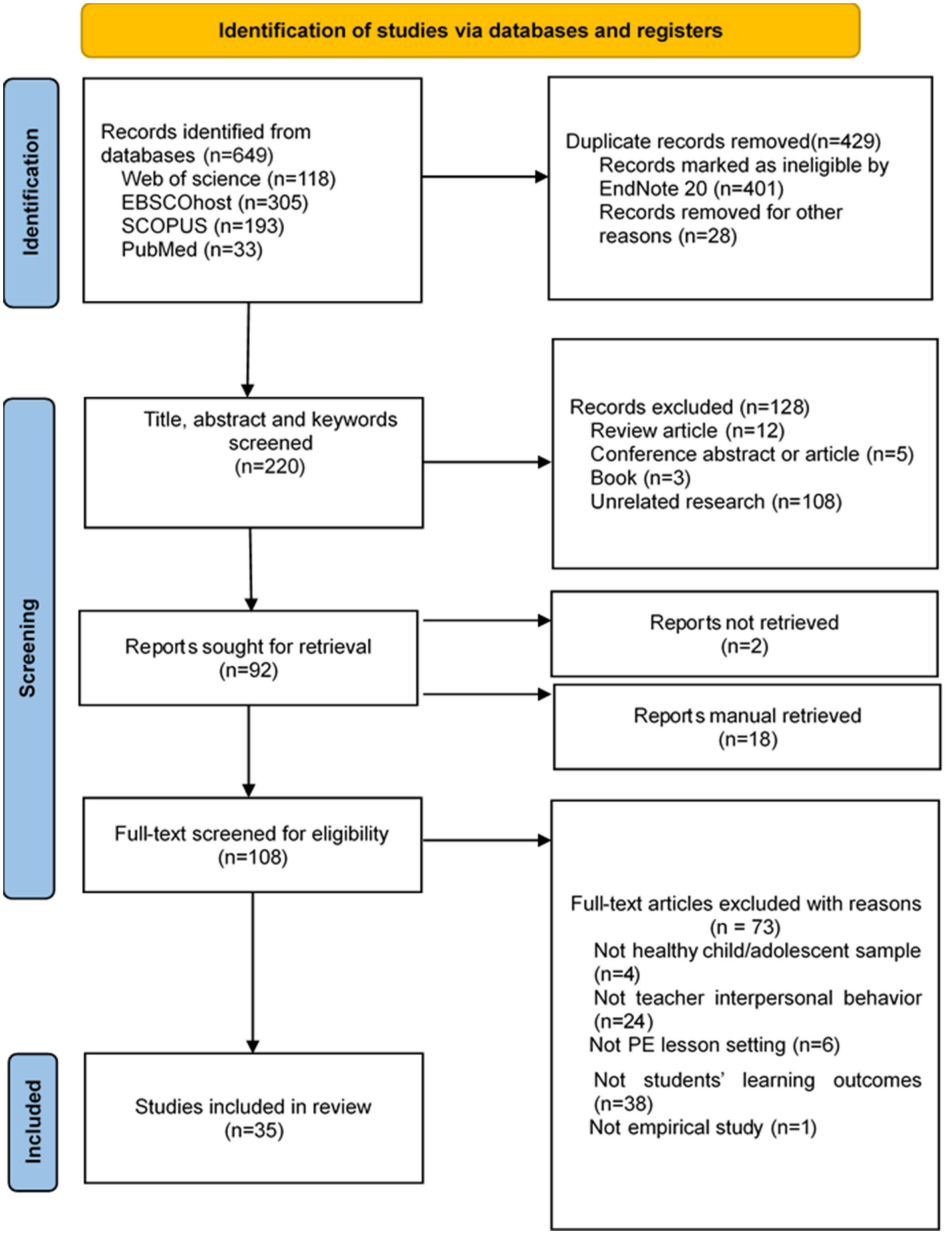
Flow diagram of literature search results.

After removing duplicate studies, a screening process was initiated. The title, abstract, and keywords were screened by two researchers. Subsequently, the two researchers independently screened the full text for eligibility. During the selection process, we consulted a third researcher to discuss any discrepancies (Figure 1).

### 2.5. Data extraction

The data extracted from the source comprised descriptive research information (authors, year of publication, country of publication, sample size, stage of education, and age range), study design, measures of the varies, reliability, and validity evidence of the measurement tools, as well as the primary outcomes from statistical analysis focused on the relationships between two variables and the effect on learning in PE (Table 1).

**Table 1:** Included studies’ characteristics.

Author/year/country	Participants (sample size/school stage/age range)	Study design	Varies	Measures of the varies	Reliability or validity	Main results	Effect on learning in PE
Van Doren et al. (2021) Belgium	302; grade 8 to 10; 11–16 years	cross-sectional study	students’ perceptions of teachers’ motivating style; students’ situational motivation in PE	Behavioral Regulations in PE Questionnaire (BRPEQ); Teacher as Social Context Questionnaire (TASCQ); Psychologically Controlling Teaching (PCT)	BRPEQ: Reliability (autonomous motivation: 0.85; controlled motivation: 0.76; amotivation: 0.66); TASCQ: Reliability (autonomy-support: 0.71; structure: 0.69; relatedness-support: 0.78) PCT: Reliability (0.77)	The relationship between instructors’ encouraging styles and students’ physical activity levels was not significant; however, the relationship between teachers’ controlling styles and students’ physical activity levels was substantial and unfavorable on the class level; Teachers’ motivating styles were significantly and positively related to autonomous motivation, controlled motivation, and amotivation at the student level; Teachers’ controlling styles were significantly and positively related to controlled motivation and amotivation.	physical activity levels; students’ situational motivation
Petiot and Desbiens (2022) France	419(231 girls and 188 boys); grades 6 to 12; mean age 14.28 years	cross-sectional study	teacher’s interpersonal behaviors; emotion	Differential Emotions Scale (DES) of Izard; Questionnaire on Teacher Interaction (QTI)	Construct validity (acceptable)	Teacher’s interpersonal behaviors was relation with emotion.	emotion
Moreno-Murcia et al. (2018) Spain	416(187 girls and 229 boys); the high school; 16–18 years	cross-sectional study	controlling teaching behaviors; importance of PE; students’ global intrinsic motivation; physical activity intention; life satisfaction; level of physical activity	Controlling Coach Behavior Scale (CCBS); Behavioral Regulation in Sport Questionnaire (BRSQ); Scale of Importance of PE(SIPE); Intention to be Physically Active Scale (IPAS); Scale of Life Satisfaction (SLS); Habitual Physical Activity Questionnaire (HPAQ)	CCBS: Reliability (0.94) Construct validity (good) BRSQ: Reliability (0.89) Construct validity (good) SIPE: Reliability (076) Construct validity (acceptable) IPAS: Reliability (0.81) Construct validity (good) SLS: Reliability (0.83) Construct validity (good) HPAQ: No	Global intrinsic motivation was negatively correlated with controlling teaching methods; however, there was a positive correlation between intrinsic motivation and life satisfaction, which was influenced by perceptions of the value of physical education, intentions to engage in physical activity, and levels of regular physical activity.	intrinsic motivation; perceived importance of PE; intentions to be physically active; physical activity level
Moreno-Murcia et al. (2022) Spain	717 (359 girls and 358 boys); 13–19 years	longitudinal study	autonomy support; needs satisfaction; intrinsic motivation; enjoyment; intention; habitual physical activity	Spanish version of the Learning Climate Questionnaire (SLCQ); Spanish version of the Psychological Need Satisfaction in Exercise Scale (SPNSES); Spanish version of the Behavioral Regulation in Sport Questionnaire (SBRSQ); Spanish version of the Intrinsic Satisfaction in Sport Questionnaire (SISSQ); Intention to be Physically Active Scale, Spanish version (IPASS); Habitual Physical Activity Questionnaire, Spanish version (HPAQS)	SLCQ: Internal consistency reliability (acceptable) SPNSES: Internal consistency reliability (good) Construct validity (acceptable) SBRSQ: Internal consistency reliability (good) SISSQ: Internal consistency reliability (good) Construct validity (acceptable) IPASS: Internal consistency reliability (good) Construct validity (acceptable) HPAQS: No	Autonomy support →Needs satisfaction → Intrinsic motivation → Enjoyment → Intention → Habitual Physical activity	habitual Physical activity
Valero-Valenzuela et al. (2021) Spain	618 (317 girls and 301 boys); the primary and secondary school; 10–14 years old	cross-sectional study	academic controlling motivation; self-esteem	Academic motivation scale (EME-E); Physical self-perception profile (PSPP)	EME-E: Internal consistency reliability (introjected regulation: 0.82; external regulation: 0.78; demotivation: 0.81) PSPP: Internal consistency reliability (0.77)	Academic controlling motivation negatively and significantly predicted self-esteem. It was essential to avoid controlling motivation to promote the development of a positive self-perception in students.	self-esteem
Fin et al. (2019) Spain	200 (105 girls and 95 boys); elementary education; 11–13 years old	longitudinal study	autonomy support; control support; basic psychological needs; motivation; physical activity enjoyment	The Learning Climate Questionnaire (LCQ); The Controlling Teacher Questionnaire (CTQ); Basic Psychological Needs in Physical Education (NPBEF); Perceived Locus of Causality Questionnaire (PLOCQ); Physical Activity Enjoyment Scale (PACES)	LCQ: Reliability (0.91) Construct validity (acceptable) CTQ: Reliability (0.86) Construct validity (acceptable) NPBEF: Reliability (autonomy:0.73 competence:0.71; relatedness:0.85) PLOCQ: Internal consistency reliability (intrinsic motivation: 0.81; identified regulation: 0.76; introjected regulation: 0.76; external regulation: 0.69; amotivation: 0.74) PACES: Reliability (0.91)	The downward pattern was associated with more negative enjoyment and a controlling style, while the upward pattern with more positive enjoyment and autonomy support.	physical activity enjoyment
Liu et al. (2017) Hong Kong, China	623; grades 7–10; 11–17 years	cross-sectional study	perceived autonomy support; perceived controlling behaviors; psychological needs satisfaction; psychological needs frustration; psychological well-being; ill-being	Health Care Climate Questionnaire (HCCQ); Psychological Control in Teaching Scale (PCTS); Psychological Needs Satisfaction Scale in PE (PNSSPE); Psychological Needs Thwarting Scale in PE (PNTSPE); Subjective Vitality Scale (SVS); Negative Affect Scale (NAS)	HCCQ: Reliability (0.84) PCTS: Reliability (0.73) PNSSPE: Reliability (autonomy: 0.81; competence: 0.83; relatedness: 0.85) PNTSPE: Reliability (autonomy: 0.82; competence: 0.79; relatedness: 0.83) SVS: Reliability (0.88) NAS: Reliability (0.85)	Autonomy support from teachers was significantly correlated to student need satisfaction and need frustration; Perceived controlling behavior was significantly correlated with need frustration; The indirect impact of perception of autonomy support by need frustration on subjective and negative affect vitality was significant; The indirect effect of perceived controlling behavior through need frustration on negative affect and subjective vitality was significant.	student need satisfaction and need frustration; psychological well-being and ill-being
Emeljanovas et al. (2020) Lithuania	222; grades 9–10; 14-18 years	cross-sectional study	teachers’ controlling behaviors; autonomy support; physical activity	Controlling Coach Behaviors Scale (CTBS); Sport Climate Questionnaire (SCQ); Accelerometers (Tri-axis Acti Trainer Activity Monitor)	CTBS: Reliability (rewards: 0.68; negative control: 0.86; threatening: 0.74; personal excessive control: 0.80) SCQ: Reliability (0.81)	For girls, autonomy support was the predictor of the time spent in MVPA in PE class but only until variables of teachers’ controlling behaviors were added, and only personal excessive controlling was positively related to MVPA in PE class; Threatening was significantly connected to PA in PE class while autonomy support was not significantly related to MVPA among boys; Teachers’ controlling behaviors variables added 4–5% to the total variance explaining MVPA in PE class.	MVPA
Koka (2014) Estonia	395 (222 girls and 173 boys); the high school; 12–16 years	longitudinal study	perceived autonomy support; health-related quality of life; self-esteem	Sport Climate Questionnaire (SCQ); Pediatric Quality of Life Inventory TM 4.0 (Peds QLTM 4.0) Generic Core Scales; Physical Self-Description Questionnaire (PSDQ)	SCQ: Reliability(good) Peds QLTM 4.0: Reliability(good) PSDQ: Construct validity(acceptable)	Students’ overall well-being (global physical self-esteem, global self-esteem, and health-related quality of life) was impacted by their perception of the PE teacher’s autonomy support.	physical self-esteem; global self-esteem; health-related quality of life
De Meyer et al. (2014) Belgium	702; grades 7–12; mean age 14.43 years	cross-sectional study	teachers’ controlling behaviors; students’ motivation for PE	Scale for Controlling Teaching Behavior (SCT); Scale for Students’ Perceptions of Controlling Teaching (SSPCT); Scale for Students’ Perceptions of Autonomy-supportive Teaching Behavior (SSPAT); Behavioral Regulations in Physical Education Questionnaire (BRPEQ)	SCT: Reliability (0.73) SSPCT: Reliability (0.88) SSPAT: Reliability (0.79) BRPEQ: Reliability (autonomous motivation: 0.89; controlled motivation: 0.86; amotivation: 0.81)	Controlled motivation, not amotivation, was substantially associated with observed controlling teaching style; The perceived autonomy-supportive teaching style and the students’ autonomous motivation were unrelated to the controlling teaching style that was observed; Perceived controlling teaching behavior had an effect on controlling motivation and amotivation.	motivation
Coterón et al. (2020) Spain	644; the middle school; 12–16 years	cross-sectional study	students’ behavioral engagement; students’ perceived autonomy support	Students’ Behavioral Engagement Scale (SBES); Students’ Perceived Autonomy Support Questionnaire (SPASQ)	SBES: Reliability (0.83) SPASQ: Reliability (0.87)	Students’ behavioral engagement in physical education classrooms was highly correlated with their perception of autonomy support.	behavioral engagement
Langdon et al. (2019) America	4 PE teachers and 140 students; the high school; mean age 41.25,14.90	cross-sectional study	autonomy-supportive teaching;	audio record	Reliability (0.83)	Such actions had the advantage of more directly impacting student motivation and improving skill acquisition.	motivation; skill learning.
Jung and Choi (2016) UK	46 (24 boys and 22 girls); eighth-grade; 1 PE teacher; 10 years experience	longitudinal study	indirect teaching behavior	audio record; interview		The teaching behaviors had a significant impact on the social and moral development of the students, and it might affect how they felt about PE and their PE teachers.	social and moral development; perception of PE and PE teachers
Aibar et al. (2021) Spain	1,118 (569 girls and 549 boys); the high school; mean age 14.11 years.	cross-sectional study	perception teachers’ need-supportive behavior; novelty satisfaction in PE; satisfaction of basic psychological needs in PE; intention of physically active	Questionnaire of Basic Psychological Needs Support in Physical Education (QBPNSPE); Basic Psychological Needs in Exercise Scale (BPNES); Novelty Need Satisfaction Scale (NNSS); Theory of Planned Behavior Questionnaire (TPBQ)		The satisfaction of basic psychological needs and novelty were positively predicted by students’ perceptions of autonomy, competence, and relatedness support from PE teachers and positively predicted intention of physically active.	novelty satisfaction in PE; satisfaction of basic psychological need in PE; intention of physically active
Koka and Hein (2006) Estonia	302 (169 boys and 133 girls); grades 6–10; 11–17 years	longitudinal study	perceived teachers’ positive feedback; perceived threat to sense of self	Perceived Teacher’s Feedback Questionnaire (PTFQ); Physical Education Learning Environment Scale (PELES)	PTFQ: congeneric and discriminant validity (good) PELES: congeneric and discriminant validity (good)	Threat perceptions of students and perceived positive feedback had a negative correlation.	perception of threat to sense of self in PE class
Koka and Hein (2005) Estonia	638 (370 girls and 268 boys); the middle and high school; 14-18 years	cross-sectional study	perceptions of teacher’s feedback; sport motivation	Modified Perceptions of Teacher’s Feedback Scale (MPTFS); Sport Motivation Scale (SMS)	MPTFS: Reliability (good) construct validity(good) SMS: Reliability (good) Construct validity (good)	The effective predictor of students’ intrinsic motivation in PE was perceived favorable feedback; The perceived teacher’s nonverbal feedback did not affect students’ motivation.	students’ intrinsic motivation in PE
Hutmacher et al. (2020) Luxembourg	1,877 (922 girls and 955 boys); the elementary and secondary schools; 10–23 years	longitudinal study	need support; motivation in PE	Need Support Scale (NSS); Revised Perceived Locus of Causality Scale (PLOC-R)	PASSES: Reliability (autonomy support: 0.82; competence support: 0.83; relatedness support: 0.81) Construct validity (good) PLOC-R: Reliability (amotivation: 0.80; external regulation: 0.71; introjected regulation: 0.68; identified regulation: 0.85; intrinsic motivation: 0.82)	Internal motivation and identified regulation were predicted by students’ perceptions of the PE teacher’s autonomy support, relatedness support and the competence support was the strongest predictor.	intrinsic motivation and identified regulation
Haerens et al. (2018) Belgium	647 (446boys and 201 girls); 8th grade; mean age 13.27 years	cross-sectional study	perceived autonomy teaching behaviors; controlling teaching behaviors; students’ experiences of need satisfaction and frustration; motivation; controlled non-participation	six positively worded items from Teacher as Social Context Questionnaire (TASCQ); 7-item Scale for Psychologically Controlling Teaching (PCT); Basic Need Satisfaction and Frustration Scale (BNSNF); Behavioral Regulations in Physical Education Questionnaire (BRPEQ) 8-item Scale of Controlled Motivated Non-Participation	TASCQ: Reliability (0.77) PCT: Reliability (0.86) BNSNF: Reliability (need satisfaction: 0.79; need frustration: 0.84) BRPEQ: Reliability (autonomous: 0.85; controlled motivation: 0.82; amotivation: 0.74) 8-item Scale: Reliability (0.93)	Students perceived high-autonomy support had the lower levels of need frustration, controlled motivation, amotivation, and controlled non-participation than those perceived high-control behaviors; Compared to the high-high group or the high-control group, the low-low group showed lower levels of need frustration, controlled motivation, amotivation, and oppositional defiance, but greater levels than the high-autonomy group (with the exception of controlled motivation and controlled non-participation).	motivation; need satisfaction and frustration
Koka and Sildala (2018) Estonia	419 (246 girls and 173 boys); grades 10–12; mean age 17.22 years	cross-sectional study	Perceived teachers’ controlling behaviors; Amotivation in PE;	Controlling Teacher Behaviors Scale (CTBS); Amotivation Inventory in Physical Education (AI-PE)	CTBS: Reliability (acceptable) Discriminant validity (acceptable) AI-PE: Reliability (good) Discriminant validity(good)	Boys showed more strength in the association between perceived teachers’ controlling behavior to praise and amotivation, while girls showed more strength in associations of perceived negative conditional regard and intimidating behavior with amotivation.	amotivation
Gairns et al. (2015) Spain	374 (374 girls); grades 7–10; mean age 13.36 years	cross-sectional study	Students’ perception of teachers; Motivation; Social anxiety;	Perceived Relatedness Support Questionnaire (PRSQ); Perceived Locus of Causality Scale (PLOCS); Social Anxiety Questionnaire (SAQ)	PRSQ: Validity (0.94) SAQ: Reliability (0.93)	When students were believed to be abilities, were connected to, respected by, understood by teachers, they had more autonomous motivation for PE. The link of teacher-derived relatedness support and students’ social anxiety was indirect.	autonomous motivation; social anxiety
Hein et al. (2015) Estonia	602 (293 girls, 309 boys); the 12–16 years	cross-sectional study	Perceived teachers’ controlling behavior; Anger	Controlling Coach Behaviors Scale (CCBS); Modified Aggression Scale (MAS)	CCBS: Validity (good) MAS: Validity (good)	Through perceived psychological needs. Students’ perception of negative actions had a significant indirect impact on anger and bullying behaviors	anger and bullying behaviors
Hein and Caune (2014) Estonia, Latvia	727 (439 girls and 288 boys); the high school; 14–18 years	cross-sectional study	Perceived teachers’ autonomy support; Motivation; Effort of students; Physical self-esteem	Perceived Locus of Causality Scale (PLOCS); Effort Subscale of the Intrinsic Motivation Inventory; PSDQ Scale	PLOCS: Reliability (intrinsic motivation: 0.89; identified regulation: 0.87; introjected regulation: 0.63; external regulation subscales 0.71)	Students’ effort perception and motivation were both directly correlated with perceived autonomous support from a teacher. Students perceived teachers’ autonomy support had an indirect effect on their perceived effort and physical self-esteem though need satisfaction of autonomy and motivation.	motivation; effort of students; physical self-esteem
Leo et al. (2020) Spain	1,120 (561girls and 559 boys); grades 5–11; 10–17 years	cross-sectional study	Perceived teaching behavior; Motivation; Engagement; Physical Activity Intentions	Teaching Interpersonal Style Questionnaire in Physical Education (TISQ-PE); The Questionnaire of Motivation in Physical Education Classes (CMEF); Engagement part of the Engagement Versus Disaffection with Learning Scale (EVDLS); Single-item Scale	TISQ-PE: Reliability (perceived need support teaching style: 0.91; perceived need thwarting teaching style: 0.89) Validity (acceptable) CMEF: Reliability (0.86) Validity (acceptable) EVDLS: Reliability (0.87) Validity (acceptable)	Physical activity engagement and intentions were positively correlated with perceived need-supportive teaching when needs were satisfied and self-motivation was present, and negatively correlated with perceived need-thwarting teaching when needs were frustrated and amotivation was present.	physical activity engagement and intentions
Haerens et al. (2015) Belgium	499 (280 girls and 219 boys); the secondary school; mean age 15.76 years	cross-sectional study	Autonomy-supportive and controlling teaching; Need satisfaction and need frustration Motivational outcomes in PE	Teacher As Social Context Questionnaire (TASCQ); Psychologically Controlling Teaching scale (PCT); Basic Psychological Need Scale and Need Frustration Scale (BPNSNF); Behavioral Regulations in Physical Education Questionnaire (BRPEQ)	TASCQ: Reliability (0.79) Validity (acceptable) PCT: Reliability (0.82) Validity (acceptable) BPNSNF: Reliability (0.87) Validity (good) BRPEQ: Reliability (autonomous motivation: 0.91; controlled motivation: 0.81; amotivation: 0.79) Validity (acceptable)	Perceived autonomy support was related primarily to autonomous motivation, with need satisfaction mediating this association, whereas perceived controlling teaching was related primarily to controlled motivation and amotivation, through need frustration.	motivation
García-González et al. (2023) Spain	1,107 (563 girls and 544 boys); the secondary school; mean age 14.12 years	cross-sectional study	Perceived competence-supportive teaching style; Perceived internally and externally controlling teaching style; Need satisfaction and frustration; Motivation	Basic Psychological Needs Support Questionnaire in Physical Education (BPNSQ); Controlling Teaching Scale for Physical Education (CTS-PE); Basic Psychological Needs in Exercise Scale (BPNES) and Basic Psychological Need Satisfaction and Frustration scale (BPNSNFS); Perceived Locus of Causality Scale (PLOC)	BPNSQ: Validity (good) CTS-PE: Validity (good) BPNES and BPNSNFS: Validity (good) PLOC: Validity (acceptable)	The competence-support positively related to need satisfaction and autonomous motivation, external and internal control positively related to need frustration, controlled motivation, amotivation; the “high competence-support-low control” profile was the most adaptive, while the “low competence-support-very high control” profile was the most maladaptive.	motivation
Van Doren et al. (2023) Belgium	904 (46.7% boys and 53.3% girls); the secondary school; mean age 13.23 years	cross-sectional study	Students’ perceptions of teachers’ Styles; Students’ need-based experiences; Students’ motivation for PE	Teacher as Social Context Questionnaire (TASCQ) and Psychologically Controlling Teaching (PCT); Basic Psychological Need Satisfaction and Need Frustration Scale (BPNSNF); Behavioral Regulations in Physical Education Questionnaire (BRPEQ)	TASCQ: Reliability (autonomy support: 0.76; structure: 0.69; relatedness support: 0.79) PCT: Reliability (0.79) BPNSNF: Reliability (need satisfaction: 0.80; need frustration: 0.83) BRPEQ: Reliability (intrinsic motivation: 0.89; identified regulation: 0.76; introjected regulation: 0.64; external regulation: 0.67; amotivation: 0.71)	Teachers’ autonomy-supportive style was positively related to students’ intrinsic motivation, showed a trend toward a positive relation with students’ identified regulation, and showed a trend toward a negative relation with students’ amotivation. Teachers’ structuring style was positively related to students’ intrinsic motivation and identified regulation, while being negatively related to students’ amotivation. Teachers’ controlling style showed an unexpected negative relation with students’ introjected regulation. Teachers’ chaotic style was negatively related to students’ identified regulation and positively to students’ amotivation.	motivation
Borghouts et al. (2021) Netherlands	612 (277 girls and 335 boys); the secondary school; mean age 14 years	longitudinal study	Teacher behavior; Motivational regulation; Psychological need satisfaction and frustration; Achievement goal orientation; Perceived mastery and performance climate	Teacher behavior: video recording; Behavioral Regulations in Physical Education Questionnaire (BRPEQ); Basic Psychological Need Satisfaction and Frustration Scale (BPNSFS); 2× 2 Achievement Goal in Physical Education Questionnaire (2× 2 AGPEQ); Motivational Climate Scale for Youth Sports (MCSYS)		Students’ achievement goal orientation decreased, while the controlled motivation (trend), amotivation, autonomy frustration (trend) and relatedness frustration increased. Other variables at the student level were insignificant.	achievement goal orientation; motivation
Tilga et al. (2020a) Estonia	1,031 (583 girls and 448 boys); the secondary school; mean age 13.39 years	cross-sectional study	Teachers’ interpersonal behavior; Students’ need satisfaction and need frustration; Students’ health-related quality of life	Multidimensional Perceived Autonomy Support Scale for Physical Education (MD-PASS-PE) and Controlling Coach Behaviors Scale (CCBS); Basic Psychological Need Satisfaction and Need Frustration Scale (BPNSNF); Pediatric Quality of Life Inventory 4.0 Generic Core Scale (PedsQL TM 4.0)	MD-PASS-PE: Validity (acceptable) CCBS: Validity (acceptable) BPNSNF: Validity (acceptable) PedsQL TM 4.0: Validity (acceptable)	Students’ perceptions of autonomy support from teachers was positively associated with health-related quality of life through need satisfaction. Students’ perceptions of controlling behavior from teachers was negatively related with health-related quality of life through need frustration.	health-related quality of life
Tilga et al. (2019b) Estonia	1,042 (589 girls and 453 boys); the secondary school; 12–15 years	longitudinal study	Perceived teachers’ controlling behavior; Perceived need frustration; Health-related quality of life	Controlling Coach Behaviors Scale (CCBS); three need frustration subscales from the basic psychological need satisfaction and frustration scale (BPNSFS); PedsQL TM 4.0		The effect of perceived teachers’ controlling behavior on health-related quality of life was mediated by need frustration.	health-related quality of life
Tilga et al. (2019a) Estonia	321 (175 girls and 146 boys); the secondary school; 11–15 years	longitudinal study	Multidimensional autonomy-supportive behavior; Multidimensional controlling behavior; Perceived need satisfaction and need frustration; Intrinsic motivation	MD-PASS-PE; CCBS; BPNSNF; PLOC	MD-PASS-PE: Reliability (good) CCBS: Reliability (acceptable) BPNSNF: Reliability (good) PLOC: Reliability (0.96)	A web-based autonomy-supportive intervention program for PE teachers significantly increased students’ perception of all autonomy-supportive teacher behaviors and students’ need satisfaction and significantly decreased students’ perception of teacher intimidation behaviors and students’ autonomy frustration, but was no significant effects on intrinsic motivation.	intrinsic motivation
Koka (2013) Estonia	330 (194 girls and 136 boys); the secondary school; mean age 13.74 years	longitudinal study	Perceived teaching behaviors; Motivational regulations	Leadership Scale for Physical Education (LSPE) and Perceptions of the Teacher’s Feedback questionnaire (PTF); Behavioral Regulations in Exercise Questionnaire (BREQ)	LSPE and PTF: Validity (acceptable) BREQ: Validity (acceptable)	The perceived teaching behaviors, such as democratic behavior, autocratic behavior, positive general feedback, and students’ autonomous motivation were reciprocally related over time; prior perceived social support and teaching and instruction were related to higher levels of subsequent autonomous motivation and lower levels of controlled motivation and not the reverse.	motivation
Koka et al. (2021) Estonia	705 (384 girls and 321 boys); the secondary school; mean age 13.65 years	cross-sectional study	Perceptions of teachers’ autonomy-supportive behavior; Psychological need satisfaction; Novelty need satisfaction; Intrinsic motivation	MD-PASS-PE; BPNSFS; Novelty Need Satisfaction Scale (NNSS); PLOC	MD-PASS-PE: Reliability (cognitive autonomy support: 0.90; procedural autonomy support: 0.86; organizational autonomy support:0.88) Validity (good) BPNSFS: Reliability (autonomy need satisfaction: 0.93; relatedness need satisfaction: 0.95; competence need satisfaction: 0.95) Validity (good) NNSS: Reliability (0.82) Validity (good) PLOC: Reliability (0.96) Validity (good)	Perceived cognitive autonomy support from teachers was indirectly related to students’ intrinsic motivation via the need satisfaction for competence and autonomy; Perceived organizational autonomy support was indirectly related to intrinsic motivation only via the need satisfaction for autonomy, whereas perceived procedural autonomy support was indirectly related to intrinsic motivation only via the need satisfaction for novelty.	intrinsic motivation
Tilga et al. (2020b) Estonia	592 (314 girls and 278 boys); the secondary school; mean age 13.58 years	cross-sectional study	Perception of teachers’ autonomy-supportive behavior; Perception of teachers’ controlling behavior; Perceived need satisfaction; Intrinsic motivation	MD-PASS-PE; CCBS; BPNSNF; PLOC	MD-PASS-PE: Reliability (good) CCBS: Reliability (acceptable) BPNSNF: Reliability (good) PLOC: Reliability (0.96)	The higher levels of controlling behavior did attenuate the indirect effect of perceived autonomy-supportive behavior on intrinsic motivation through need satisfaction.	intrinsic motivation
Hein et al. (2018) Estonia	3,108 (1,564 girls and 1,544 boys); the secondary school; mean age 14.27 years	cross-sectional study	Perceived autonomy support behavior; Need satisfaction; Effort; Physical self-esteem	Perceived Locus of Causality Scale (PLCS); Perception of Need Satisfaction Scale (PNSS); Intrinsic Motivation Inventory (IMI); PSDQ		Students’ perceived autonomy support from the teacher was directly related to effort and indirectly via autonomous motivation, whereas physical self-esteem was related indirectly.	effort; physical self-esteem
De Meyer et al. (2016) Belgium	925 (398 girls and 527 boys); the secondary school; mean age 15.80 years	cross-sectional study	Controlling teaching; Student motivation;	Psychologically Controlling Teaching Scale (PCTS); Behavioral Regulations in Physical Education Questionnaire (BRPEQ)	PCTS: Reliability (externally controlling teaching: 0.78; internally controlling teaching: 0.71) Validity (acceptable) BRPEQ: Validity (acceptable)	Controlling teaching (and internally controlling teaching in particular) is related to maladaptive motivational outcomes.	motivational outcomes

### 2.6. Quality assessment

Utilizing a technique established by the Agency for Healthcare Research and Quality (AHRQ), the quality of the included manuscripts was evaluated, which examined cross-sectional studies. The evaluation criteria were evaluated using 11 items, including data sources, inclusion criteria, observation time, and evaluators**’** subjective factors. Included studies were scored with it (“Yes” = 1 point, “No” or “unclear” = 0 point). The full score of the scale was 11 points, and scores ≥ 8 were classified as high quality, 4**–**7 as moderate quality, and ≤ 3 as low quality (Hu et al., 2015). For longitudinal studies, we assessed the quality of articles using the Newcastle-Ottawa Scale (NOS; Wells et al., 2000), which was assessed using three aspects: study population selection, intergroup comparability, and outcome measurement, including eight items. The full score was 9 points, and scores ≥ 8 were classified as high-quality, 5**–**7 as moderate quality, and < 5 as low-quality (Li et al., 2014).

## 3. Results

### 3.1. Study selection results

By searching the subject terms, 649 articles were retrieved from the electronic databases. First, the retrieved literature was imported into Endnote, a literature management software, and 429 duplicates were removed. Next, by screening the title, abstract, and keywords, we excluded irrelevant articles (108), review articles (12), conference abstracts or articles (5), and books (3). After searching the full text of reports, two manuscripts were not found, and 18 more resent important manuscripts were added by manual retrieval and reading the full text of the remaining 96 papers. Ultimately, this review included 35 studies, including ten longitudinal studies and 25 cross-sectional studies (Figure 1).

### 3.2. Study characteristics

**Figure 2:**
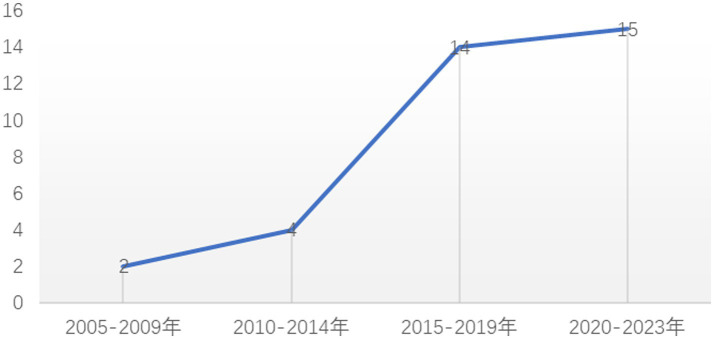
The number of published articles from 2005 to 2023.

**Figure 3:**
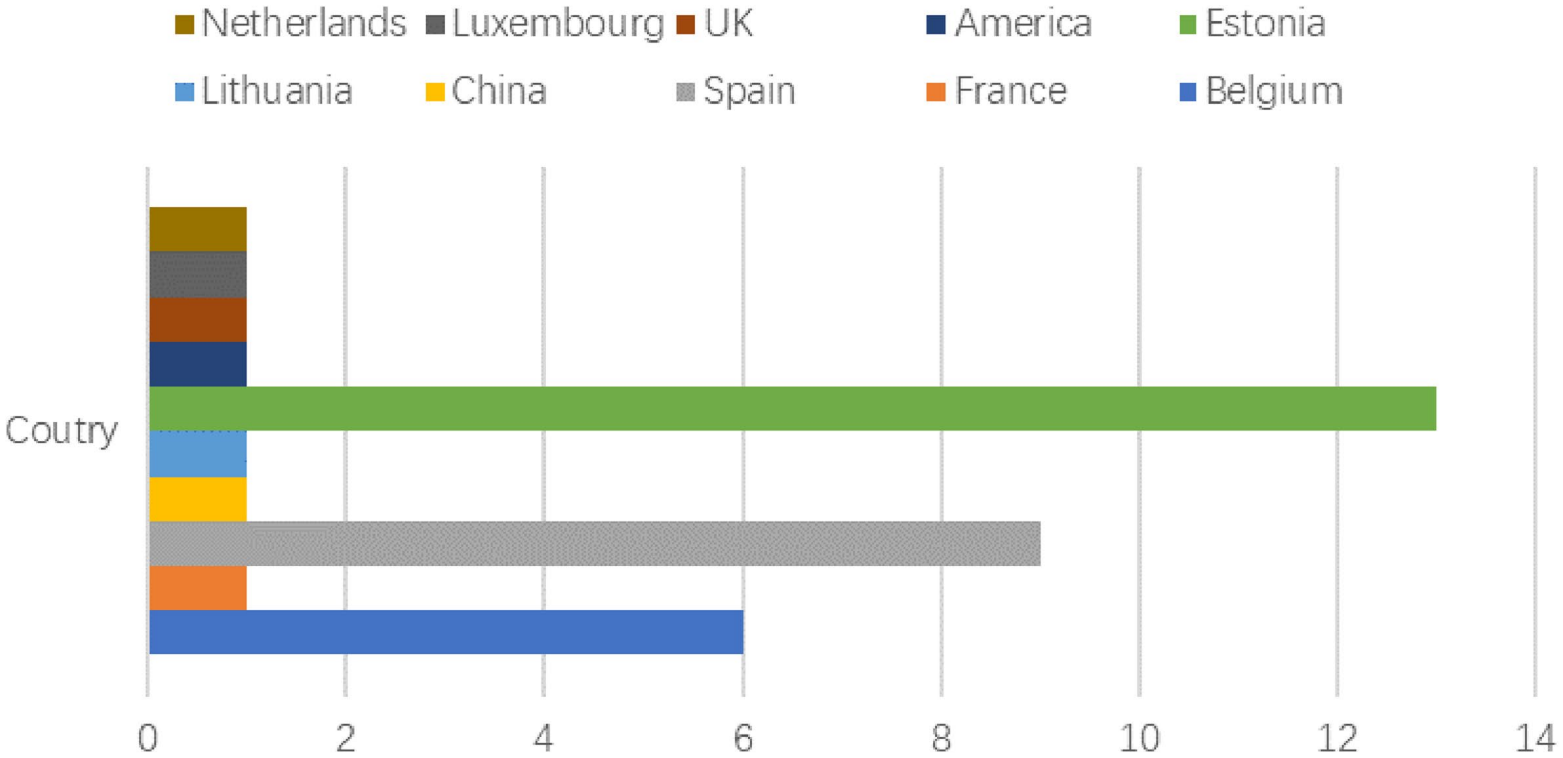
The number of published articles on different countries.

The 35 articles that made up the systematic review (Table 1) were all published between 2005 and 2023, and since 2015, more research has been conducted on PE teachers’ interpersonal behaviors than before (Figure 2). The total sample of students was 24,444, from ten countries, including Belgium (6), France (1), Spain (9), China (1), Lithuania (1), Estonia (13), the United States (1), the United Kingdom (1), Luxembourg (1), and Netherlands (1) (Figure 3). Most of the 35 studies were centered on secondary school students, and only two study was conducted in an elementary school; the age of students ranged from 10 to 18 years.

Regarding study design, 25 of the included studies adopted a non-experimental cross-sectional design, and ten adopted a longitudinal design. The exposure factor was perceived teachers’ interpersonal behavior, which was measured primarily using a subjective self-report questionnaire. In the present review, 33 studies used self-report scales, and two studies were qualitative, using audio recording techniques and interviews to obtain data. Except for two studies, the publications provided detailed accounts of the measurement instrument’s reliability and validity, with most achieving a high quality level. The outcome variables explored by the researchers, primarily grounded in self-determination theory, concentrated on PE learning motivation (20), physical activity levels, or physical activity intention (7), and several studies explored the effect on emotions (5) and students’ psychological well-being (7).

According to the quality assessment results of the included studies (Tables 2, 3), seven studies were classified as high-quality, 27 studies as moderate-quality, and no low-quality studies were identified.

**Table 2:** Quality assessment of included cross-section studies.

	1	2	3	4	5	6	7	8	9	10	11	Total score	Quality
Van Doren et al. (2021)	1	1	1	1	1	1	0	1	1	1	0	9	H
Petiot and Desbiens (2022)	1	1	1	1	1	0	0	0	0	1	0	6	M
Moreno-Murcia et al. (2018)	1	1	1	1	1	1	0	0	0	1	0	7	M
Liu et al. (2017)	1	1	1	1	1	1	0	1	1	1	0	8	H
Emeljanovas et al. (2020)	1	1	1	1	1	1	0	0	0	0	0	5	M
De Meyer et al. (2014)	1	1	1	1	1	1	0	1	0	1	0	7	M
Coterón et al. (2020)	1	1	1	1	1	1	0	0	0	0	0	5	M
Langdon et al. (2019)	1	1	1	1	1	1	0	0	0	0	0	5	M
Aibar et al. (2021)	1	1	1	1	1	0	0	0	1	1	0	7	M
Koka and Hein (2005)	1	1	1	1	1	1	0	0	1	1	0	7	M
Haerens et al. (2018)	1	1	1	1	1	1	0	1	1	1	0	8	H
Koka and Sildala (2018)	1	1	1	1	1	1	0	0	1	1	0	7	M
Gairns et al. (2015)	1	1	1	1	1	1	0	1	1	0	0	8	H
Hein et al. (2015)	1	1	1	1	1	1	0	0	0	0	0	6	M
Hein and Caune (2014)	1	1	1	1	1	1	0	0	0	0	0	6	M
Leo et al. (2020)	1	1	1	1	1	1	0	1	0	1	0	8	H
Valero-Valenzuela et al. (2021)	1	1	1	1	1	1	0	0	0	1	0	7	M
García-González et al. (2023)	1	1	1	1	1	1	0	0	0	0	0	6	M
Van Doren et al. (2023)	1	1	1	1	1	1	0	0	0	0	0	6	M
Tilga et al. (2020a)	1	1	1	1	1	1	0	0	1	1	0	8	M
Koka et al. (2021)	1	1	1	1	1	1	1	0	1	1	0	9	H
Tilga et al. (2020b)	1	1	1	1	1	1	0	0	1	0	0	7	M
Hein et al. (2018)	1	1	1	1	1	1	0	1	0	0	0	7	M
De Meyer et al. (2016)	1	1	1	1	1	1	0	0	0	0	0	6	M

**Table 3:** Quality assessment of included longitudinal studies.

	1A	1B	1C	1D	2	3A	3B	3C	Total score	Quality
Koka (2014)	1	1	0	1	0	1	1	1	6	M
Jung and Choi (2016)	1	1	1	1	0	1	1	1	7	M
Koka and Hein (2006)	1	1	0	1	0	1	1	1	6	M
Hutmacher et al. (2020)	1	1	1	1	0	1	1	1	7	M
Fin et al. (2019)	1	1	1	1	0	1	1	1	7	M
Moreno-Murcia et al. (2022)	1	1	1	1	0	1	1	1	7	M
Borghouts et al. (2021)	1	1	1	1	0	1	1	1	7	M
Tilga et al. (2019b)	1	0	1	1	0	1	1	1	6	M
Tilga et al. (2019a)	1	1	1	1	2	1	1	1	9	H
Koka (2013)	1	1	1	1	0	1	1	1	7	M

### 3.3. The effect of perceived teachers’ interpersonal behavior on students’ learning in PE

#### 3.3.1. The effect of perceived teachers’ interpersonal behavior on students’ learning motivation in PE

Twenty studies investigated PE’s impact on students’ learning motivation. The classification of motivation and the division of sub-dimensions in these studies were mostly based on the self-determination theory (SDT). Deci and Ryan (1985) distinguished three different types of motivation, intrinsic motivation, extrinsic motivation, and amotivation, which were used to explain the reasons for an individual’s behavior (Deci and Ryan, 1985). Furthermore, based on the classic definitions of intrinsic and extrinsic motivations, organismic integration theory which was a subtheory within SDT, described four different types of motivation under the broad category of extrinsic motivation, external regulation, introjected regulation, identified regulation, and integrated regulation (Ryan and Deci, 2000). The four types of extrinsic motivation vary in the amount of autonomy, and the least autonomy form is external regulation, followed by introjected regulation. These two forms of motivation are also called controlled extrinsic motivation. On the more autonomy side are identified regulation, and integrated regulation, which are also called autonomous extrinsic motivation (Standage et al., 2005). Indeed, in some analyses, researchers have categorized intrinsic motivation, identified regulation, and integrated regulation as autonomous motivations (Cheon et al., 2012; Haerens et al., 2015, 2018; Van Doren et al., 2021). Once an individual’s intrinsic motivation is stimulated, without any external rewards or constraints, one’s behavior is completely self-regulated, and the participation in activities is motivated by his or her own interest, displaying a strong sense of will. In contrast, extrinsic motivation is various modes of regulation that have an instrumental character. That is, extrinsic motivation is distinguished from intrinsic motivation by whether an individual’s behavior in engaging in an activity is driven by a separable, extrinsic factor (e.g., reward, punishment, and threat).

In these studies, the researchers found that the students’ perceptions of their teachers’ interpersonal behaviors had a direct or indirect impact on their motivation. In general, the autonomous motivation of students was positively correlated with teachers’ supporting behaviors, while the controlling motivation and amotivation of students were positively correlated with teachers’ controlling behaviors. Hein and Caune (2014) concluded that self-determined motivation was directly impacted by the perception of teachers’ autonomy support (Hein and Caune, 2014). Hutmacher et al. (2020) discovered that PE teachers’ autonomy support, related support, and competence support all strongly influenced their students’ identified regulation and intrinsic motivation (Hutmacher et al., 2020). While, Van Doren et al. (2023) found teachers’ autonomy-supportive style was positively related to students’ intrinsic motivation, showed a trend toward a positive relation with students’ identified regulation (Van Doren et al., 2023), so that the relationship between identified regulation and teachers’ supportive behaviors is not uncertain. In Haerens et al. (2018), they compared the motivation of students in a high-autonomy support group to that of students in a high-control group. They discovered that students in the high autonomy support group had the lowest levels of controlled motivation, amotivation, and controlled non-participation (Haerens et al., 2018). At the same time, controlling teaching (and internally controlling teaching in particular) was related to maladaptive motivational outcomes (De Meyer et al., 2016), the higher levels of controlling behavior did attenuate the indirect effect of perceived autonomy-supportive behavior on intrinsic motivation through need satisfaction (Tilga et al., 2020b). However, one study concluded when teachers’ autonomy-support increased, students’ achievement goal orientation decreased while the controlled motivation, amotivation, autonomy frustration and relatedness frustration increased (Borghouts et al., 2021). And another web-based intervention program for PE teachers did not lead to significantly higher student perceptions of intrinsic motivation (Tilga et al., 2019b).

Langdon et al. (2019) presented the advantages of autonomy-supportive behaviors that directly impact students’ motivation in a typical PE class using context analysis to ascertain how and when teachers engage in such behaviors (Langdon et al., 2019). The indirect path may be that the effect perceived autonomy support on autonomous motivation for PE, mediated by need satisfaction (Koka, 2014), and perceived controlling teaching was related primarily to controlled motivation and amotivation, through need frustration (Haerens et al., 2015). Furthermore, the high support-low control profile was the most adaptive, while the low support-high control profile was the most maladaptive (García-González et al., 2023).

One study evaluated the effect of gender on the relationship between perceived teachers’ controlling behavior and PE amotivation; it concluded that girls had higher amotivation and were stronger than boys in associations with teachers’ perceived intimidating behavior and negative conditional regard, while boys showed more strength than girls in the association between perceived teachers’ controlling use of praise and amotivation (Koka and Sildala, 2018). Van Doren et al. (2021) revealed that teachers’ motivational styles were significantly and positively connected to autonomous motivation, controlled motivation, and amotivation (Van Doren et al., 2021). Similarly, other studies also discovered that global intrinsic motivation was adversely correlated with controlling teaching styles (Moreno-Murcia et al., 2018) and the best indicator of students’ intrinsic motivation for PE was perceived as favorable feedback (Koka and Hein, 2005). In detail, perceived cognitive autonomy support from teachers was indirectly related to students intrinsic motivation via the need satisfaction for competence and autonomy; Perceived organizational autonomy support was indirectly related to intrinsic motivation only via the need satisfaction for autonomy, whereas perceived procedural autonomy support was indirectly related to intrinsic motivation only via the need satisfaction for novelty (Koka et al., 2021).

Additionally, a qualitative research (De Meyer et al., 2014) used video technology to record PE teachers’ behaviors to assess their classroom management in a real-world setting and discovered that observed controlling teaching behavior had a positive effect on controlled motivation but was unrelated to autonomous motivation and amotivation. Perceived controlling teaching behavior was related to controlled motivation and amotivation. Gairns et al. (2015) concluded that when students felt that their PE teacher had a high level of confidence in their abilities, they reported being more autonomously motivated toward PE. Additionally, analyses showed that when students felt connected to, appreciated by, and understood by their teachers, they reported being more autonomously motivated to participate in physical activity (Gairns et al., 2015). Furthermore, the relationship perceived teaching behaviors (such as democratic behavior, autocratic behavior, positive general feedback) and students’ autonomous motivation may be reciprocal (Koka, 2013).

#### 3.3.2. The effect of perceived teachers’ interpersonal behavior on students’ learning emotion in PE

Five studies offered insight into students’ learning emotions in PE class. On the one hand, one study explored positive learning emotions. Petiot and Desbiens (2022) examined students’ emotional experience and the perception of teachers’ interpersonal behaviors based on the socio-cognitive theory of emotion in PE. They concluded that male students experienced positive emotions, which were associated with female teachers’ positive interpersonal behaviors, whereas female students experienced less positive emotions, perceived teachers’ interpersonal behaviors less favorably, and expressed less positive appreciation of PE (Petiot and Desbiens, 2022). By contrast, researchers have also explored negative learning emotions. Liu et al. (2017) found an indirect impact of both perceived autonomy support and perceived controlling behavior on negative affect through need frustration (Liu et al., 2017). Gairns et al. (2015) demonstrated an indirect pathway linking teacher-derived relatedness support with students’ social anxiety, and the intermediate agencies were relatedness need satisfaction and controlled motivation (Gairns et al., 2015). Teachers’ negative behaviors could affect students’ angry feelings significantly and indirectly in PE learning through the mediating variable of psychological need thwarting (Hein et al., 2015). One longitudinal study also discovered that the downward pattern was associated with more negative enjoyment and a controlling style, while the upward pattern with more positive enjoyment and autonomy support (Fin et al., 2019).

#### 3.3.3. The effect of perceived teachers’ interpersonal behavior on students’ psychological well-being in PE

Seven studies have explored the effect of teachers’ interpersonal behaviors on students’ psychological well-being factors, including subjective vitality, self-esteem, and social and moral development. Liu et al. (2017) revealed that students’ perceptions of autonomy-supportive teaching behaviors had a significant effect on their subjective vitality, which reflected psychological well-being and was mediated by need satisfaction (Liu et al., 2017). Koka (2014) conducted a tracking study, collecting data three times over 6 months, and proposed that perceptions of PE teachers’ autonomy support were essential antecedents of students’ overall well-being variables, such as physical self-esteem, global self-esteem, and health-related quality of life (Koka, 2014). And the indirect pathways students’ perceptions of teachers’ behaviors affected health-related quality of life through need satisfaction or need frustration (Tilga et al., 2019b, 2020a). From another perspective, perceived positive general teacher feedback and perception of threat to sense in PE class had a negative correlation and students felt bad about himself indicating perception of threat to his self-esteem (Koka and Hein, 2006). Jung and Choi (2016) conducted an ethnographic case study, observing 8 months in two coed eighth-grade PE classes, and concluded that teaching behavior had a significant impact on the social and moral growth of students (Jung and Choi, 2016). Students’ perceived autonomy support from the teacher was related with physical self-esteem (Hein et al., 2018), and controlling motivation negatively and significantly predicted self-esteem (Valero-Valenzuela et al., 2021). It was essential to avoid controlling motivation to promote the development of a positive self-perception in students.

#### 3.3.4. The effect of perceived teachers’ interpersonal behavior on students’ physical activity levels

Seven studies explored the association between students’ levels of physical activity in PE classes and perceived teachers’ interpersonal behaviors. Van Doren et al. (2021) investigated the relationship between students’ perceptions of PE teachers’ motivating style and students’ device-based physical activity levels during PE and found that only teachers’ autonomy support and students’ physical activity levels had a significant positive relationship; the relatedness support and structure sub-dimensions did not (Van Doren et al., 2021). Another study evaluated the effect of gender on the association between perceived teachers’ behavior and the degree of physical activity. Emeljanovas et al. (2020) discovered that for girls, the amount of time spent in MVPA in class was predicted by autonomy support, and only the personal excessive controlling component was positively related to MVPA. For boys, threatening was significantly associated with physical activity, while autonomy support was not significantly associated with MVPA in PE classes. In general, teachers’ controlling behavior variables added 4–5% to the total variance explaining MVPA in PE class (Emeljanovas et al., 2020). Additionally, researchers conducted studies in PE classes to observe how teachers’ behaviors impacted students’ learning engagement (Coterón et al., 2020; Leo et al., 2020), which reflected students’ distinct competences, such as endeavor, attention, and perseverance, which might raise the level of physical activities (Kirk, 2005), revealing a significant relationship between students’ behavioral and emotional engagement and their perception of autonomy support in PE classes.

The relationship between teachers’ interpersonal behaviors and physical activity levels may be indirect, and the connection was made through some mediating variables. Moreno-Murcia et al. (2018) concluded that the relationship between the controlling teaching style and students’ global intrinsic motivation was mediated by the perceived importance of PE in class, which was negatively correlated with intrinsic motivation. Global intrinsic motivation, in turn, predicted students’ perception of the importance of PE in class and how much physical activity they engaged in Moreno-Murcia et al. (2018). Aibar et al. (2021) discovered that basic psychological needs and novelty satisfaction of students were predicted by perceptions of teachers’ autonomy, competence, and relatedness support. Additionally, basic psychological needs and novelty satisfaction predicted the intention to engage in physical activity (Aibar et al., 2021). Moreno-Murcia et al. (2022) carried a longitudinal design to explore the effect autonomy support on habitual PE of adolescents, and found the indirect influence path was “autonomy support → needs satisfaction → intrinsic motivation → enjoyment → intention → habitual physical activity,” that may be provide a new direction for this filed study (Moreno-Murcia et al., 2022).

## 4. Discussion

This study’s purpose is to provide the first systematic review of studies on the link between perceived teachers’ interpersonal behaviors and students’ PE learning. By screening, identifying, describing, and synthesizing the empirical studies in this area, we expected to shed light on the importance of teachers’ interpersonal behaviors for students. Based on the findings, we have discussed the main results of the review. In doing so, we delineate areas that need to be focused on to improve the learning effects in teaching interventions in PE classes and identify the most significant challenges to future research.

### 4.1. The effect of perceived teachers’ interpersonal behavior on students’ learning in PE

#### 4.1.1. The effect of perceived teachers’ interpersonal behavior on students’ learning motivation in PE

According to the above results, researchers have shown that students’ motivation is affected by perceived teachers’ interpersonal behaviors, either directly or indirectly. In general, teachers’ supportive behaviors, which involve autonomy, competence, and relatedness supports, were related to students’ autonomous motivation (Langdon et al., 2019; Hutmacher et al., 2020; Van Doren et al., 2021). That is, when students feel that teachers respect their suggestions, encourage initiative speaking, give students freedom, and show patience (Haerens et al., 2015); give constructive feedback, set rules with rationales, and adjust physical activities to students’ learning situations (Jang et al., 2010); and express affection in interacting with students, enhance cooperative and interdependent learning tasks, and create a tolerant learning environment (Cox and Williams, 2008), their inner autonomous motivation resources are gradually cultivated. Additionally, teachers’ controlling behaviors are corrected by students’ controlling motivation and amotivation (De Meyer et al., 2014; Van Doren et al., 2021). Control style teachers tend to take disciplinary action, criticize students who do not meet expectations, adopt normative feedback, ignore students’ development of ability or individual differences, and remain cold and alienated from students (Reeve, 2006; Sheldon and Filak, 2008; Bartholomew et al., 2009). This may lead to students’ lack of learning motivation or even amotivation. The mechanism may be that teachers’ supportive teaching behaviors satisfy students’ basic psychological needs, while teachers’ controlling teaching behaviors frustrate children’s basic psychological needs (Leo et al., 2020). However, one study found when teachers’ autonomy-support increased, students’ controlled motivation, amotivation, autonomy frustration and relatedness frustration increased, the reason maybe was teachers’ performance climate decreased through the intervention approaches.

Furthermore, it is crucial to reduce teachers’ controlling behaviors and enhance their interpersonal behaviors, which could lead to negative learning outcomes in PE such as anger, boredom, anxiety, and unhappiness (Ntoumanis, 2001; Standage et al., 2005; Moreno-Murcia et al., 2018). The findings showed that teachers’ behaviors involved different pathways to student learning, including supporting and controlling teaching actions, in which the former was a “bright” way, the latter was a “dark” way (Haerens et al., 2011).

#### 4.1.2. The effect of perceived teachers’ interpersonal behavior on students’ learning emotion in PE

In this review, our findings showed a clear association between the perception of teachers’ interpersonal behaviors and students’ learning emotions in PE class. When students perceive less support from teachers, they feel less appreciated, express fewer positive emotions, and even feel anxiety and shame (Petiot and Desbiens, 2022). The social situation is the origin of emotion, according to the socio-cognitive theory of emotion (Lazarus, 1991). If students receive an unfavorable stimulus environment from teachers, they will evaluate teachers’ interpersonal behaviors, which then cause negative emotions.

Additionally, the present study demonstrated an indirect pathway link evidencing indirect pathways of teacher-student connections (Gairns et al., 2015; Liu et al., 2017). The most probable indirect effect mechanism was students’ psychological needs, and whether that was satisfied or not determined the nature of students’ emotions.

#### 4.1.3. The effect of perceived teachers’ interpersonal behavior on students’ psychological well-being in PE

We found evidence that teachers’ interpersonal behaviors affected students’ psychological well-being factors in PE. When students perceived higher autonomy support, they experienced greater psychological well-being because their psychological needs were satisfied; when students perceived more controlled behaviors, they experienced more psychological well-being because their psychological needs were frustrated (Liu et al., 2017). Further, PE teachers’ autonomy support was an antecedent to students’ health-related quality of life, with the relationship mediated only by the psychological needs subdimension-need satisfaction for relatedness (Koka, 2014). The reason behind these results may be that teachers’ autonomy support is more strongly correlated with students’ relatedness needs than other aspects (Hagger et al., 2009). Thus, the literature provided inconsistent and insufficient evidence regarding this pathway where teachers’ interpersonal behaviors affected psychological well-being. Research should further explore this aspect of the influence mechanism in the relationship.

#### 4.1.4. The effect of perceived teachers’ interpersonal behavior on students’ physical activity levels

The findings here did not clarify whether perception of teachers’ interpersonal behaviors was associated with students’ physical activity levels. There is little evidence to support this relationship. For teachers’ supportive behavior, only autonomy support was significantly and positively linked to the level of students’ physical activity in PE class; the other sub-dimensions did not have significant relationships. Regarding teachers’ controlling behavior, only two studies confirmed a significant and negative relationship with pupils’ physical activity level at the class level (Kirk, 2005; Van Doren et al., 2021). When students perceived more autonomy support from the teacher, they engaged in the PE class actively and volitionally; when students perceive more teacher control, the physical activity time spent on PE lessons was reduced, which was contrary to the traditional assumption that students must be actively in the control of PE teachers.

Thus, it was more likely that teachers’ interpersonal behaviors did not directly affect physical activity level. The present study provided evidence to show the indirect pathways through mediating variables generating connection. The most likely effect mechanism was students’ personal psychological tendencies, such as intrinsic motivation, basic psychological needs, and novelty satisfaction (Moreno-Murcia et al., 2018; Aibar et al., 2021), which predicted whether students would be physically active. Research in this area must be strengthened.

### 4.2. Limitations and directions for future research

To the best of our knowledge, this is the first systematic review of studies on teacher interpersonal behaviors in the PE context, including the bright side (teachers’ supportive behaviors) and dark side (teachers’ controlling behaviors) of teacher behaviors, which are the two sides of the same coin. However, this review has some limitations. First, the included studies were published in English, which can lead to the omission of important literature published in other languages. Second, most included studies used self-reported measures that are a form of self-attribution, which could lead to subjective rather than objective responses. Some studies have proposed exploring implicit questionnaires, which can reduce the bias of social approval reaction and fraud in the participants’ answers. Additionally, the breadth and depth of research on teachers’ interpersonal behaviors is limited, and the influence mechanisms affecting learning in PE situations remains unclear. For example, studies focused on students in secondary school, and the applicability of the findings in primary education and higher education is unknown. Currently, more studies have explored the effect of teachers’ interpersonal behaviors on students’ motivation according to SDT, while few studies have explored other aspects of academic life.

The most important direction for further study is to examine the impact of teachers’ interpersonal behavior on the learning process or learning results in PE classes, and then examine the indirect influence mechanism through which a connection occurs. A key focus is teachers’ interpersonal behavior and students’ engagement in PE that is an index of students’ high-quality involvement (Skinner et al., 2009),and how PE teachers’ behaviors predict students’ engagement. For example, Leo et al. (2020) showed needs satisfaction and in turn quality of motivation mediate the relations of need-supportive teaching to PE engagement (Leo et al., 2020). Especially behavioral engagement, which embodies various abilities, efforts, and concentration of students, which could improve their level of physical activity (Kirk, 2005). Moreover, it is widely acknowledged the teachers’ interpersonal behaviors affect students’ learning in PE. It is necessary to understand what can lead to teachers to adopt the style of behaviors and develop the effective intervention programs. Therefore, we could explore the specific classification of teacher behaviors and identify the antecedents of teachers’ behavior styles to intervene teachers to improve the practice. For example, Ahmadi et al. (2023) used a Delphi method to create a classification of teacher behaviors consistent with SDT, and made a list of teacher motivational behaviors (Ahmadi et al., 2023). Escriva-Boulley et al. (2021) found the antecedents of primary school teachers’ need-supportive and need-thwarting styles in PE were the pressures from below, above, and within (Escriva-Boulley et al., 2021).

Another direction for future study is to enrich research on PE’s impact on students’ learning emotions. According to the cognitive theory of emotion, this puts more emphasis on the connection between social conditions and emotion. In this case, teachers’ interpersonal behavior creates a situational atmosphere that induces positive or negative classroom learning emotions in students. In fact, negative emotions not only undermine students’ learning but also reduce their activity level (Petiot and Desbiens, 2022). Thus, the present study only found the mechanism with SDT, and other applicable theories should be promulgated. One important referable theory is the Model for Interpersonal Teacher Behavior, which seeks to verify the connection between the different types of teachers’ interpersonal behaviors and students’ cognitive and emotional learning outcomes in the classroom context (Brekelmans et al., 1989; Wubbels et al., 2006), which could contribute to academic achievement.

The subjects of the included studies were teenage students aged 10**–**18 years. Future research could expand the age range, explore younger children under 10 years old and older youth (college students) over 18 years old to enhance our understanding of how perceived teachers’ interpersonal behavior affects physical education learning for all student levels from primary school to university.

Regarding study design, most studies adopted a cross-sectional approach; longitudinal and rigorously designed research is lacking. This requires expanding such research directions and advancing better quality studies, as the time sequence factor is the necessary condition to verifying the variables’ causality. Therefore, we could explore the future direction of the link between teachers’ interpersonal behaviors and students’ physical learning. Finally, future studies could explore multi-level models to investigate how teachers’ behaviors affect students’ PE learning.

## 5. Conclusion

This research is the first systematic review of the studies that examined the effect teachers’ interpersonal behavior in class has on students’ learning in the PE situation. The results showed that teachers’ interpersonal behaviors are linked with students’ learning motivation, learning emotions, physical activity levels, and psychological well-being. Clearly, perceived teachers’ supportive behaviors impact students’ autonomous motivation, and perceived teachers’ controlling behaviors affect students’ controlling motivation and amotivation. The influencing mechanism may be that teachers’ interpersonal teaching behaviors satisfy or frustrate students’ basic psychological needs according to SDT. Another main issue is that it remains unclear whether perception of teachers’ interpersonal behaviors impact students’ physical activity levels, and more evidence is needed. Additionally, we found that perceived teachers’ interpersonal behaviors have an effect on students’ PE learning emotions. In other words, perceiving teachers’ supportive behaviors triggers students’ positive learning emotions; by contrast, perceiving teachers’ controlling behaviors stimulates students’ negative learning emotions. Finally, there is insufficient evidence that teachers’ interpersonal behaviors have an effect on students’ psychological well-being.

We suggest that substantial additional research is needed, including verifying indirect influence mechanisms, involving mediating factors, and moderating factors. Moreover, the results show that teachers’ controlling behavior can hinder and block students’ learning processes, leading to undesired learning outcomes. Accordingly, encouraging teachers’ support behavior and reducing teachers’ control behavior can be effective in enhancing the quality of PE teaching and promote students’ academic performance.

## Data availability statement

The datasets presented in this study can be found in online repositories. The names of the repository/repositories and accession number(s) can be found in the article/Supplementary material.

## Author contributions

JC: conceptualization and methodology. LT: formal analysis and write the manuscript. All authors contributed to the article and approved the submitted version.

## Funding

This research was funded by the Social Science Foundation of Jiangxi Province, China, grant number 20JY50.

## Conflict of interest

The authors declare that the research was conducted in the absence of any commercial or financial relationships that could be construed as a potential conflict of interest.

## Publisher’s note

All claims expressed in this article are solely those of the authors and do not necessarily represent those of their affiliated organizations, or those of the publisher, the editors and the reviewers. Any product that may be evaluated in this article, or claim that may be made by its manufacturer, is not guaranteed or endorsed by the publisher.
